# Miniaturized Spoof Plasmonic Antennas with Good Impedance Matching

**DOI:** 10.3390/nano13010136

**Published:** 2022-12-27

**Authors:** Yi Ren, Jingjing Zhang, Xinxin Gao, Xin Zheng, Le Peng Zhang, Tie Jun Cui

**Affiliations:** 1 Institute of Electromagnetic Space, Southeast University, Nanjing 210096, China; 2 State Key Laboratory of Millimeter Waves, Southeast University, Nanjing 210096, China; 3 Frontiers Science Center for Mobile Information Communication and Security, Southeast University, Nanjing 210096, China

**Keywords:** spoof surface plasmon polaritons, miniaturized antenna, impedance matching

## Abstract

The ability of spoof surface plasmon polaritons (SSPPs) to confine electromagnetic fields in a subwavelength regime enables the design of miniaturized antennas. However, the impedance matching scheme for miniaturized spoof plasmonic antennas has not been studied systematically. In this paper, we propose a general method in the antenna design based on SSPPs, providing a feasible solution to impedance matching at the feeding point of miniaturized spoof plasmonic antennas. To verify the method, a prototype of a planar spoof plasmonic dipole antenna is simulated, fabricated and measured, of which the dipole arm length is reduced by 35.2% as compared with the traditional dipole antenna. A peak gain level of 4.29 dBi and the radiation efficiency of about 94.5% were measured at 6 GHz. This general method can be extended to solve the impedance matching problem in the design of other spoof plasmonic devices.

## 1. Introduction

Metamaterials have attracted lots of interest and have been widely used in electromagnetics and antenna engineering [1], such as in spatial electromagnetic filtering [2], beam steering [3], near-field transformation [4], absorbers [5] and so on. Spoof surface plasmon polaritons (SSPPs), also known as plasmonic metamaterials, inherit the properties of field confinement and local field enhancement from natural surface plasmon polaritons (SPPs) by decorating metal surfaces with subwavelength corrugations [6,7,8,9]. Thus far, various devices have been proposed based on SSPPs, such as filters [10,11,12,13], couplers [14,15], amplifiers [16,17,18], harmonic generators [19,20,21], sensors [22,23,24] and lens [25,26]. The integration of spoof plasmonic devices/components into traditional circuits requires conversion from guided waves to spoof plasmonic waves. In order to achieve a high conversion efficiency, good impedance matching between SSPPs and guided waves is vital. Therefore, various conversion structures from traditional transmission lines, such as the microstrip (MS) and coplanar waveguide (CPW), to spoof plasmonic waveguides have been proposed [27,28,29], where a good transmission performance was demonstrated experimentally. 

Additionally, conventional antenna prototypes can take on an altogether new aspect with spoof plasmonic structures, which realize the conversion between SSPP waves and free-space waves [30,31,32,33,34,35,36]. Spoof plasmonic structures can serve as not only the feeding structures but also radiating elements, favoring the miniaturization of spoof plasmonic antennas [37]. Generally, gradient corrugations applied to achieve impedance matching in spoof plasmonic antennas increase the antenna profile and hinder further miniaturization [38,39]. A frequency-dependent characteristic impedance extraction method was proposed as an alternative approach for the conventional gradient-corrugation strategy to achieve the compact transformation of SSPP waveguides, but it is not suitable for SSPP antenna designs [40]. Recently, the impedance matching concept in an analogy to radio frequency (RF) technology was introduced in optical nanocircuits to optimize the coupling between the plasmonic nanoantenna and the transmission line [41,42,43]. However, a systematic methodology for the impedance matching of miniaturized spoof plasmonic antennas remains to be investigated.

In this paper, the impedance matching of miniaturized spoof plasmonic antennas is discussed. Using the analytical fitting of standing wave patterns along the feeding transmission line, the load reflection coefficient and load impedance of spoof plasmonic antennas are available. A miniaturized spoof plasmonic dipole antenna with good impedance matching is designed and measured to verify the feasibility of the proposed method. The numerical simulations and experiments demonstrate that the spoof plasmonic dipole antenna with a low profile shows good performances in terms of the gain and radiation efficiency. This design method is not restricted to the specific case of a dipole antenna, as discussed in this article, but can be applied in the design of more general types of spoof plasmonic devices. 

## 2. Methods

**Figure 1:**
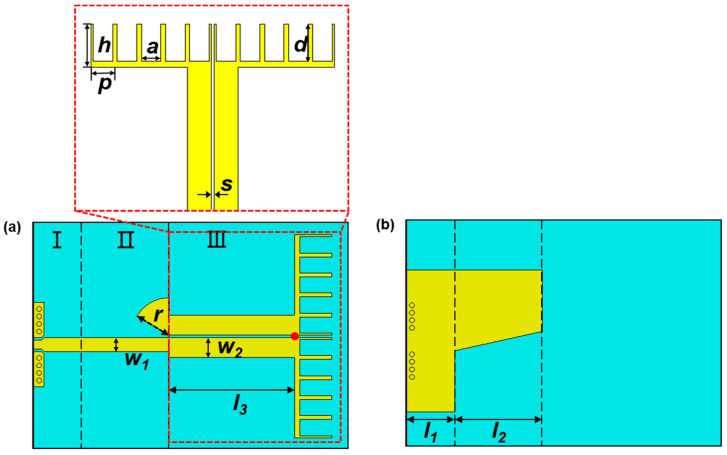
Top (**a**) and bottom (**b**) schematic diagrams of the proposed spoof plasmonic dipole antenna, where *l*_1_ = 3.8 mm, *l*_2_ = 7 mm, *l*_3_ = 10 mm, *w*_1_ = 1.14 mm, *w*_2_ = 1.6 mm, *r* = 3 mm, *s* = 0.2 mm, *p* = 1.6 mm, *h* = 3 mm, *a* = 1.3 mm, *d* = 2.6 mm.

Based on classical transmission line theory, the impedance matching between the excitation part and the radiation part of an antenna can be analyzed and quantified. For example, an equivalent circuit of a simple dipole antenna attaching the coplanar stripline (CPS) consists of an RF source with the source impedance Z_S_, a CPS with the characteristic impedance Z_0_ and a load with the load impedance Z_L_ representing the dipole antenna arms. The reflection coefficient between the feeding transmission line and the dipole antenna arms is governed by impedance matching, which can be optimized by adjusting the geometrical structure and dimension of the dipole antenna arms. The E-field distribution along the transmission line can be expressed as

(1) $$\left|E\right|=\left|E_{0}e^{ikx}\left(1+\Gamma_{v}e^{i2k\left(L-x\right)}\right)\right|$$

where *i* is the imaginary unit, *E*_0_ is the maximum E-field amplitude, *k* = *k*’ + *i k*” denotes the propagation constant consisting of the wave vector *k*’ and the decay constant *k*” of the propagating mode, *L* is the length of the transmission line along the propagating direction and Γ_V_ = |Γ_V_|*e^i^^θ^*^Γ^ is the complex reflection coefficient at the load where a red spot is marked in Figure 1a. 

The standing wave parameters along the transmission line are collected for the fitting procedure based on Equation (1). As a result, the maximum E-field amplitude *E*_0_, the propagation constant *k* consisting of *k*’ and *k*” and the complex reflection coefficient Γ_V_ consisting of its module |Γ_V_| and argument *θ*_Γ_ are obtained by using the curve fitting tool in the MATLAB. For more accurate Γ_V_ and faster calculation, we can beforehand remove the load to obtain a rough value of *k* which can be used in the following fitting procedure as an initial value. When energy is fully reflected, |Γ_V_| is near 1, which is a basis of judgment for the rough *k*. The proper value ranges of five unknowns in the procedure improve the accuracy. Then, a fitting procedure for the standing wave pattern on the transmission line with a load operates. Once the complex reflection coefficient Γ_V_ and characteristic impedance Z_0_ are known, the load impedance Z_L_ can be determined according to

(2) $$Z_{L}{=Z}_{0}\frac{{1+\Gamma }_{V}}{1-\Gamma_{V}}$$

Having known the load impedance of the radiation part, the impedance matching in the feeding network can be realized. Because the standing wave pattern on the transmission line can be observed experimentally by near-field scanning platform, we can adopt the proposed method to extract the realistic impedance of circuit elements. Based on the above method, with the length and width of the dipole arms changing, the minimization of reflection coefficient at the load and the optimization of impedance matching between Z_L_ and Z_0_ in the case of a simple dipole antenna can be realized. Obviously, designing the feeding network or transitions based on the load impedance also contributes to the impedance matching. 

## 3. Results and Discussion

To verify the feasibility of the proposed method, a prototype of a spoof plasmonic dipole antenna is simulated, fabricated and measured. The whole structure of a miniaturized spoof plasmonic antenna is shown in Figure 1, where the dielectric substrate is Rogers RO4003C with a dielectric constant of *ε_r_* = 3.55, loss tangent tanδ = 0.0027 and thickness of *t_s_* = 0.508 mm. Parts I and II illustrate the conversions from a coplanar waveguide with ground (GCPW) to an MS and from an MS to a CPS, which are called the GCPW-MS transition and MS-CPS transition, respectively. A solder-free connector is attached to the GCPW structure in the experiments to reduce the energy reflection between the sample and RF source. The metal ground plane on the bottom of the sample acting as a reflector can increase the antenna directivity and gain. The radial stub in part II provides a virtual short circuit and the RF continuity between the CPS conductor and MS ground plane [44]. Their dimensions are selected as *l*_1_ = 3.8 mm, *l*_2_ = 7 mm, *w*_1_ = 1.14 mm and *r* = 3 mm. In addition, part III is the core of the spoof plasmonic dipole antenna, which consists of a CPS and dipole arms based on spoof plasmonic periodic grooves. The dispersion curve of the SSPP periodic groove is obtained by using the Eigen-mode Solver of the CST Microwave Studio. The unit cell shown in Figure 1 is simulated, where the periodic boundary is set in the y-direction and the perfect electric conductor (PEC) boundaries are set in other orthogonal directions. The dispersion curve of the unit cell deviates from the light line gradually with the increase in the wavenumber, while the cut-off frequency decreases as the groove depth *d* increases [45]. The dimensions of the CPS are chosen as *w*_2_ = 1.6 mm, *l*_3_ = 10 mm and *s* = 0.2 mm. For the minimum |Γ_V_|, the physical dimensions of the periodic groove are determined as *p* = 1.6 mm, *h* = 3 mm, *a* = 1.3 mm and *d* = 2.6 mm, and *n* represents the number of periodic grooves on a single arm.

**Figure 2:**
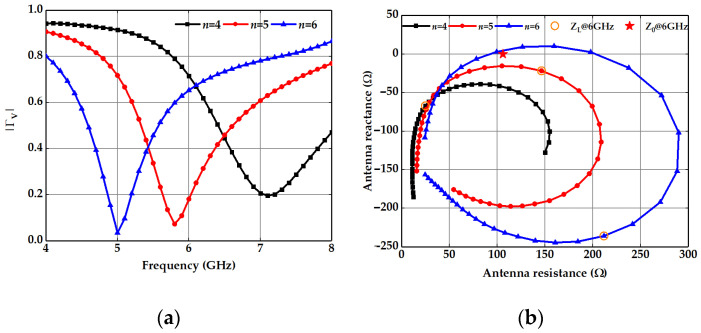
(**a**) |Γ_V_| of spoof plasmonic dipole antennas (*n* = 4, 5 and 6). (**b**) Load impedance of spoof plasmonic dipole antennas (*n* = 4, 5 and 6) in the complex plane from 4 GHz to 8 GHz. The star represents the position of Z_0_ at 6 GHz, and the circles show Z_L_ of spoof plasmonic dipole antennas (*n* = 4, 5 and 6) at 6 GHz.

Here, we optimize the structural parameters of a spoof plasmonic dipole antenna according to our proposed method so that the impedance matching can be efficiently realized at the working frequency of 6 GHz. In this case, the parameter *n* is selected in the parameter optimization procedure and full-wave electromagnetic simulations are performed using the CST MWS. A nonlinear fitting algorithm that can minimize the sum-of-squares errors is applied to the standing wave pattern along the center of the CPS transmission line when *n* = 4, 5 and 6, respectively. Thus, the complex reflection coefficient Γ_V_ is determined and its absolute value |Γ_V_| is shown in Figure 2a. It can be observed that when *n* increases from 4 to 6, the center frequency decreases from 7.1 GHz to 5.8 GHz and then to 5 GHz. Having determined the physical dimensions of the CPS and the dielectric substrate, its characteristic impedance Z_0_ is 105.9 Ω via a CPS calculator. The load impedance at the red spot of the spoof plasmonic dipole antenna is obtained via Equation (2). In Figure 2b, the load impedance Z_L_ of three spoof plasmonic dipole antennas with different antenna arm lengths is illustrated. When *n* is fixed, the load impedance shows a spiral in the complex impedance plane where Z_0_ is already marked. As the antenna arm length increases, the radius of the impedance spiral increases. The distance |Z_L_ − Z_0_| in the complex plane indicates the degree of impedance matching. The shortest distance |Z_L_ − Z_0_| at 6 GHz in Figure 2b corresponds to the minimum |Γ_V_| in Figure 2a, verifying the best impedance matching of the load impedance Z_L_ to Z_0_ at 6 GHz when *n* = 5. 

**Figure 3:**
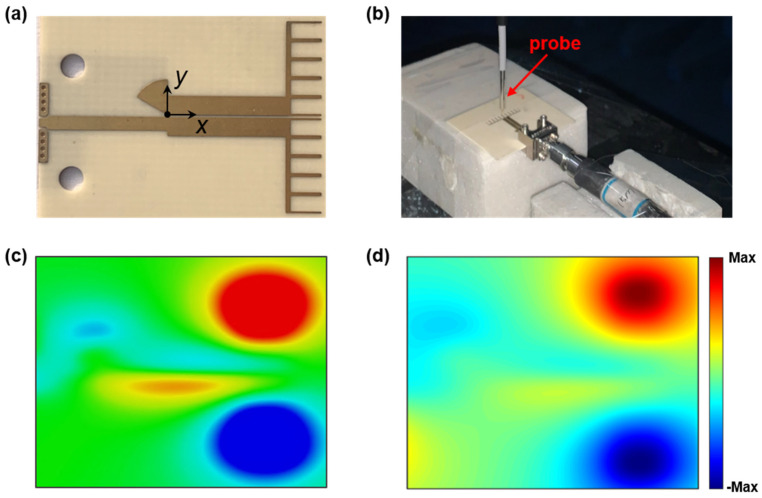
(**a**) Photograph of the sample. (**b**) Photograph of the near-field scanner. Simulated (**c**) and measured (**d**) near-field distribution of the sample at 6 GHz.

The fabricated sample for the experiments is shown in Figure 3a, where eight metal via-holes with a radius of 0.2 mm and two holes with a radius of 1 mm are tailored for a solder-free connector. Furthermore, the near-field distribution of |*E*_z_| at about 1 mm above the sample is measured at 6 GHz. The near-field scanner is composed of a vector network analyzer (VNA) and a monopole probe which can be controlled by a mechanical platform, as shown in Figure 3b. In the experiment, the input port of the sample under test is connected with one port of the VNA, while the other port of the VNA is connected with the probe in order to scan the vertical component of the E-field on the plane. Then, the field distribution on the plane can be captured by the probe. In Figure 3c,d, it is clear that the simulated and measured near-field distributions at 6 GHz are in good agreement and the reverse phase can be observed on the two dipole arms. The spoof plasmonic radiating structure is excited by the CPS successfully, and the energy is transmitted along the CPS and then radiated into the free space via the spoof plasmonic structure. 

**Figure 4:**
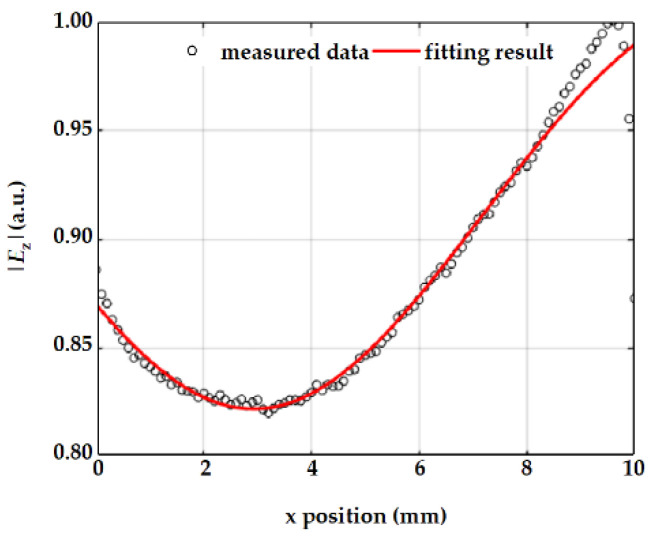
Analytical fitting to the corresponding measured |*E*_z_|.

The measured near-field along the CPS can be observed in the line scan plot in Figure 4, where the data points represent the variation in the near-field amplitude signal along the length of the CPS and the red curve is the fitting curve from a nonlinear fitting algorithm based on Equation (1). The deviation near the end of the CPS is because the analytical expression assumes a sudden termination at the end of the feeding transmission line, while in the practical test, the dipole antenna is connected to the CPS providing the field continuity. Owing to the line scan analysis, we can deduce the complex reflection coefficient via Equation (1), which favors the calculation of the load impedance using Equation (2), and validate the impedance matching method of a miniaturized spoof plasmonic antenna. Finally, the load impedance Z_L_ is calculated as 122.93 − i 1.66 Ω using the experimental data at 6 GHz when *n* = 5, while the load impedance Z_L_ in Figure 2b is 146.24 − i 21.71 Ω based on the simulated data. The difference between the simulated and measured impedance values is mainly due to the aforementioned deviation in the analytical fitting and the perturbations of the home-made near-field scanner which is small but unavoidable. In addition, the gap between two conductors is 0.2 mm while the probe has a certain volume, making it difficult to accurately mark the middle line of the CPS transmission line by sight in the experiment. The fabrication imperfections may account for the deviation between the experiment and the theory as well. 

**Figure 5:**
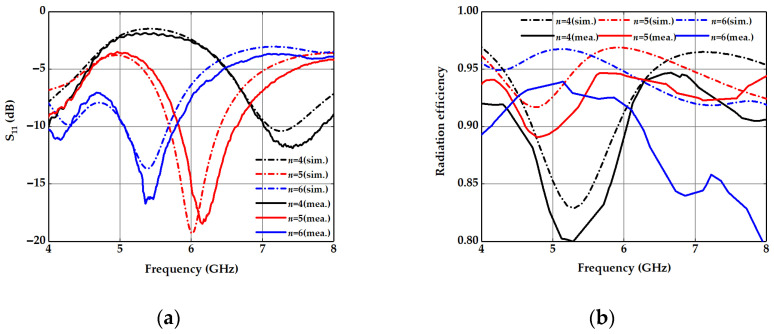
(**a**) Simulated and measured S_11_ of spoof plasmonic dipole antennas (*n* = 4, 5 and 6). (**b**) Simulated and measured radiation efficiency of spoof plasmonic dipole antennas (*n* = 4, 5 and 6).

**Figure 6:**
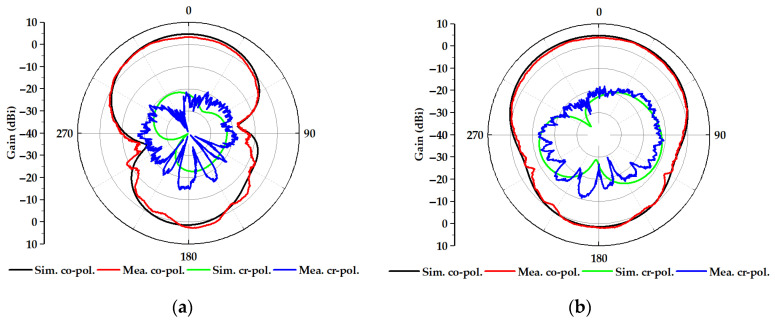
(**a**) Simulated and measured E-plane far-field patterns of the miniaturized spoof plasmonic dipole antenna (*n* = 5). (**b**) Simulated and measured H-plane far-field patterns of the miniaturized spoof plasmonic dipole antenna (*n* = 5).

In order to further demonstrate the performance of the proposed spoof plasmonic dipole antenna, the port reflection S_11_ and the radiation efficiency are simulated and measured, as shown in Figure 5. The VNA (Ceyear3672B) is used to measure these samples in the experiment. The frequency of the lowest S_11_ in the sample (*n* = 5) comes to 6.175 GHz, which is from the drift error of the VNA and the fabrication imperfections, and within the allowable error range. Meanwhile, the measured S_11_ trend of different samples coincides well with the simulated results. Based on a generalized Wheeler cap method and source-stirring [46], the measured radiation efficiency can reach 94.5% at 6 GHz, which is close to the simulated result of 96.9%. The far-field radiation patterns of a spoof plasmonic dipole antenna at 6 GHz are depicted in Figure 6. We can observe that the simulated and measured patterns in the E-plane match well with each other, with the peak gains of 4.71 dBi and 3.86 dBi, respectively. Meanwhile, the simulated and measured patterns in the H-plane are also in agreement and with the peak gains of 4.73 dBi and 4.29 dBi individually. The cross-polarization level is less than −10 dBi for both planes in all directions. These simulated and measured results show that the proposed miniaturized spoof plasmonic dipole antenna has a higher realized peak gain and a 35.2% reduction in the radiating edge dimension against the conventional dipole antenna, while its radiation patterns differ subtly from those of traditional dipole antennas in the operating frequency. Compared with the spoof plasmonic dipole antenna in [38], our proposed spoof plasmonic dipole antenna has a higher peak gain with a miniature size and high radiation efficiency, resulting from the subwavelength property of spoof plasmonics and good impedance. As compared with other slow-wave antennas, including a meander antenna [47] and spiral antenna [48], our proposed spoof plasmonic antenna is compact, conformal and suitable for monolithic microwave integrated circuits without extra longitudinal space.

## 4. Conclusions

In summary, we proposed an impedance matching method in miniaturized spoof plasmonic antennas. The near-field distribution along the transmission line linked to radiating dipole arms allows us to obtain the load reflection coefficient and load impedance by the analytical fitting of the electric field. Thus, impedance matching between the feeding transmission line and the radiating spoof plasmonic structure can be accomplished via an optimization of the structure parameters. Based on this impedance matching strategy, we have demonstrated experimentally a spoof plasmonic dipole antenna with an improved performance as compared with previous antennas, including a low profile and high efficiency. Moreover, this method has potential application prospects in various devices, in addition to the proposed spoof plasmonic antennas, and can also be extended to higher frequency ranges such as the Terahertz spectrum. 

## Data Availability

Data available on request from the authors.
